# Assessment of the Hemispheric Lateralization of Grapheme-Color Synesthesia with Stroop-Type Tests

**DOI:** 10.1371/journal.pone.0119377

**Published:** 2015-03-20

**Authors:** Mathieu J. Ruiz, Jean-Michel Hupé

**Affiliations:** 1 Grenoble Institut des Neurosciences, Institut National de la Santé et de la Recherche Médicale U836 & Université Grenoble Alpes, 38000 Grenoble, France; 2 Centre de Recherche Cerveau et Cognition, Université de Toulouse & Centre National de la Recherche Scientifique, 31300 Toulouse, France; University Zurich, SWITZERLAND

## Abstract

Grapheme-color synesthesia, the idiosyncratic, arbitrary association of colors to letters or numbers, develops in childhood once reading is mastered. Because language processing is strongly left-lateralized in most individuals, we hypothesized that grapheme-color synesthesia could be left-lateralized as well. We used synesthetic versions of the Stroop test with colored letters and numbers presented either in the right or the left visual field of thirty-four synesthetes. Interference by synesthetic colors was stronger for stimuli in the right hemifield (first experiment, color naming task). Synesthetes were also faster in the right hemifield when naming the synesthetic color of graphemes (second experiment). Overall, the lateralization effect was 7 ms (the 95% confidence interval was [1.5 12] ms), a delay compatible with an additional callosal transfer for stimuli presented in the left hemifield. Though weak, this effect suggests that the association of synesthetic colors to graphemes may be preferentially processed in the left hemisphere. We speculate that this left-lateralization could be a landmark of synesthetic grapheme-color associations, if not found for color associations learnt by non-synesthete adults.

## Introduction

Grapheme-color synesthesia is shared by 1–5% of the children and adult population [1–4]. Most grapheme-color synesthetes report that they have been associating the same specific colors to letters for as long as they can remember. However, Simner and colleagues [3] tested children for grapheme-color synesthesia in two sessions over a 12-month period (from age 6/7 to 7/8 years old), and then 3 years later [5]. They showed that children detected as synesthetes had acquired new associations between the successive sessions even though the youngest children had already learned the whole alphabetical sequence [3]. These results suggest that grapheme-color associations are acquired once the bases of reading have been mastered and that they are developed upon them. Numerous studies have shown that the processing of linguistic skills is strongly lateralized (for reviews, see [6, 7]). These skills are not exclusively processed in one hemisphere but to some degree in each: this is referred to as the degree of asymmetry or lateralization. Clinical (e.g. handedness), anatomical (e.g. the size of the planum temporale) and functional (e.g. functional MRI brain activation to linguistic material) factors can be good indicators of the degree of lateralization. These factors provide estimation that linguistic skills are dominantly processed in the left hemisphere in 90% of right-handers and 70% of left-handers [8–10]. Because the development of grapheme-color synesthesia might build upon those lateralized processes, the processing of grapheme-color synesthesia could be lateralized as well.

Indeed, two single-case studies, based mostly on phenomenology, suggested that grapheme-color synesthesia might be lateralized. Ramachandran and Hubbard [11] tested two synesthetes. One of them did not report synesthetic colors anymore for graphemes presented both beyond 11° of eccentricity and in the left visual field (LVF). Synesthetic colors were still reported in the right visual field (RVF), a result compatible with a preferential coding of synesthetic colors in the left hemisphere. However, the other synesthete did not experience synesthetic colors beyond 11° of eccentricity whatever the hemifield. Moreover, Brang and Ramachandran [12] also observed some lateralization for another synesthete, but on the other side. These results suggested possible lateralization effects in grapheme-color synesthesia, as long as inter-subject variability is taken into account. A few functional and structural neuroimaging studies also suggested brain lateralization, but with no consistency across studies. For example, Nunn and collaborators [13] suggested the lateralization of synesthetic colors in the left V4/V8 area (color region in the fusiform gyrus). However, Rouw and Scholte [14] found significant activation by synesthetic colors in the right, not the left, fusiform gyrus. Neufeld and colleagues [15] measured significant activation related to synesthesia only on the left side, but this time in the parietal cortex. Published studies have, in fact, not provided yet any clear-cut picture of the neural bases of grapheme-color synesthesia [16, 17].

In the absence of direct, neuronal, measures of brain lateralization in synesthesia, differences in response times revealed by visual half-field presentation techniques may provide some indication of brain lateralization. These techniques have been used with the Stroop task [18] to probe the lateralization of reading. As put by Brown and colleagues [19], “superficially, the basic prediction appears straightforward”: for words presented in the RVF, information “is transmitted directly to the left hemisphere” (LH) and therefore processed faster. For words presented in the LVF information is first transmitted to the right hemisphere (RH) and then needs to reach the LH for semantic processing. However, Brown et al. [19] warned that “such a straightforward prediction must be approached with caution” because of “the complexities of interhemispheric processing”. Indeed, the literature on the RVF advantage for semantic processing is far from clear-cut. For example, using a Stroop task, Brown et al. [19] did observe an RVF advantage, confirming other lateralized Stroop experiments [20]. However, Belanger and Cimino [21], in a meta-analysis of 19 lateralized Stroop studies, found no hemifield difference for interference effects. Hemifield effects, if real and to be revealed, may critically depend on the precise procedure and instructions. For example, Franzon and Hugdahl [22] measured stronger interference on a Stroop task for color-words presented in the RVF in a group of subjects instructed to be as accurate as possible, not in a group instructed to respond as fast as possible. This interesting literature seems to have waned over the last ten years with the advent of functional MRI, which allows a much more direct measure of possible lateralization effects. However, it left unresolved the possible relationship between cortical lateralization and hemifield effects for word processing (note however that effects when observed always revealed a LH, not RH, advantage; unresolved also does not mean absent). In the absence of clear-cut fMRI data about the lateralization of synesthetic colors, we return to experimental psychology to evaluate possible hemifield differences in grapheme-color synesthesia. Any hemifield difference, if observed, may constitute a starting point to consider the possible brain lateralization of synesthetic color associations. The objective of this study is yet modest, because the literature on the standard Stroop taught us that the absence of observed lateralization of the synesthetic Stroop effect would not prove the absence of brain lateralization.

We used the synesthetic version of the Stroop task to measure lateralization effects. According to Belanger and Cimino [21], Stroop tasks “require a timed, naming response to a stimulus that is presented in conjunction with a single, verbal, semantic distracter”. Here, the semantic distractor was the synesthetic color (we only chose graphemes whose synesthetic color was easy to name). Graphemes were dispatched in a color (real color hereafter) that was the same as (Congruent) or different from (Incongruent) the synesthetic color (photism hereafter) that each synesthete reported being associated with each grapheme. Subjects performed two experiments with the same stimuli. The first task was to name the real color as quickly and accurately as possible (Color Naming task hereafter). Longer response times for incongruent relative to congruent colored stimuli indicate that synesthetic colors are difficult to ignore [23, 24]. In the second task, they had to name the synesthetic color (Photism Naming task hereafter) [16, 25–27]. We presented single graphemes in the periphery of the visual field during the synesthetic Stroop task in order to reveal a possibly lateralized processing of grapheme-color synesthesia. Such lateralization could show up in two ways. First, synesthetic colors could take longer to name when graphemes are presented in the LVF, if synesthetic colors are coded in the LH (additional delay for a callosal transfer from the right retinotopic cortex). Second, under the same hypothesis, the strength of the interference by synesthetic colors in the Color Naming task could be stronger (longer response times for incongruent stimuli) for stimuli presented in the RVF (synesthetic colors processed faster have a greater chance to have the time to interfere with the Color Naming task).

To present the data, we followed the “New Statistics” guidelines proposed by Cumming [28], as recommended now in several Psychology journals, including Psychological Science [29]. We therefore present and interpret confidence intervals instead of meaningless p-values. We report p-values only for readability and comparison with previous studies in the field. However, readers should be cautious about not interpreting them [30]: when two confidence intervals are similar (large overlap), which one has the lower p-value (or whether only one of them is “significant”, for example) is utterly meaningless.

A preliminary report of these data (based on the first 22 subjects, among 34) was published in abstract form (Ruiz MJ and Hupé JM (2009) Society for Neuroscience Abstracts: 380.384).

## Materials and Methods

### Participants

We asked synesthetes who contacted us to send us a list of the letters and numbers that triggered synesthetic colors and to indicate (by precise description or print) the real color that best fitted them. When they came to the lab to perform the experiment, we did a surprise retest of their grapheme-color associations using a modified version of the “Synaesthesia Battery Test” [31]. This allowed us to check the consistency of their associations over time [32]. All synesthetes’ color choices with the Synaesthesia Battery were highly consistent with the colors they chose during the initial mail exchange, with no more than one or two differences between the two (as judged by the experimenters). Synesthetes with differences usually told us that they had several possible associated colors for that grapheme (we did not use these graphemes in the tasks). Thirty-four adult synesthetes (27 women, age range [19 52], mean = 28.5, median = 25.5; 2 left-handed, 1 ambidextrous) performed the lateralized version of the synesthetic Stroop test. They had normal or corrected to normal vision and normal color vision, as checked by the Farnsworth Lantony D-15 test, which evaluates the ability to discriminate colors. All subjects gave informed, written, consent for their participation. The first 8 subjects were recruited for a Master’s project by M.J. Ruiz. Other subjects were tested with the objective of including them later in fMRI experiments [16]. Recruitment ended (N = 34, no subjects excluded) when the planned number of subjects for the fMRI experiments was reached (Ruiz, Hupé, & Dojat, in preparation). The local ethics committee (Institutional Review Board of Grenoble) authorized the experiments (CPP 06-CHUG-23, approval dates: 10/01/2007 and 01/04/2009; CPP 12-CHUG-17, approval date 04/04/2012).

### Materials

For each synesthete, we identified which graphemes had similar synesthetic colors (see S2 Dataset for the stimuli used for each participant). We selected 4 such pairs of graphemes that 1) had easy-to-name colors: the French color words for ‘Red’, ‘Green’, ‘Blue’ and ‘Yellow’ whenever possible; This color set was achieved for 17 synesthetes; Other colors used were ‘White’, ‘Brown’, ‘Purple’, ‘Orange’ and ‘Pink’; we could not find four color categories with two graphemes having about the same color in 8 synesthetes: For one of them we used only 3 categories (25% less trials); For the other 7 synesthetes we added one (5 synesthetes) or two (2 synesthetes) colors, without changing the total number of trials (2/5 or 4/6 color categories had therefore twice less trials), 2) elicited strong synesthetic associations according to the phenomenological account by the synesthete and 3) did not start with the first letter of a color name to avoid priming effects (only three exceptions: one blue, “bleu” in French, ‘B’ for a synesthete, one yellow, “jaune”, ‘J’ for another one, and one brown, “marron”, ‘M’ for a last one). We verified that these protocol differences, inevitable due to the diversity of the synesthetes’ color associations, did not influence the results. We observed that a letter often had a similar shade of color as a digit. To increase consistency across subjects, we chose for each color category a digit and a letter, whenever possible. Graphemes were presented in the color chosen by the synesthete (congruent stimuli) or in the color chosen for another grapheme (incongruent stimuli). For each grapheme, three incongruent stimuli were generated by choosing the exact synesthetic color of one grapheme of each other pair. For example, if the synesthetic color of A was red, incongruent stimuli were green, blue and yellow “A”, colors associated to E, 3 and N by this synesthete. Graphemes subtended a maximum size of 1.5° and were presented 5° to the left or to the right of the central fixation cross. Graphemes were presented on a gray background halfway between minimum and maximum luminance capacity of the screen. Experiment and data processing were programmed in Matlab and run on a computer with Windows XP.

### Procedure

Synesthetes performed two lateralized tasks [25]. In the Color Naming task, they had to name the ‘real’ color in which the grapheme appeared on the screen (i.e. the color of the ink) while ignoring their own synesthetic color. In the Photism task, they had to name the synesthetic color of the grapheme while ignoring the physical color of the grapheme. For both tasks they had to answer as quickly as possible. If they did not name the correct color or slurred their words, they were encouraged to rapidly correct themselves. Subjects were asked to constantly fixate on the central cross. The pre-stimulus period varied between 200 and 700 ms. Graphemes appeared for only 150 ms (to prevent involuntary ocular saccades from landing on the stimulus) either in the LVF or the RVF. They were always colored, either congruently or incongruently, with respect to the photism (see *Materials* section above). After the subject answered, the experimenter launched the next trial by pressing the space bar. We applied this procedure because when subjects trigger the next trial themselves, they tend to synchronize the key press with their vocal response. This may parasite the vocal task and cause premature recording cuts. Each combination of position and congruency appeared 72 times. For incongruent stimuli, each of the three incongruent colors was used exactly 24 times. For each task, synesthetes performed 288 trials (2*2*72) in different random sequences, with a pause every 24 trials. All subjects ran the Color Naming task first. We chose the same fixed order for all subjects because we wanted to be able to compare the individual performance of synesthetes (see in the *Data analysis* section below the paragraph on the *Strength of the synesthetic association*). Subject sat in a lit room at 57 cm from the center of the screen (no chinrest) and wore a helmet microphone. Vocal responses were recorded with the software Audacity as the first track of a stereo recording. When a grapheme appeared, a black square at the bottom of the screen turned white during 10 frames. A photodiode covered this square (making it invisible to the subject) and its signal was recorded as the second track of the stereo recording, thus permitting the synchronization of vocal responses with stimulus onsets during post processing.

### Data analysis

#### Measure of Response Times

Vocal responses were processed using programs developed in Matlab. They were first synchronized to the onset of the stimuli using the amplitude of the photodiode signal. We defined the response time as the time when the absolute amplitude of the vocal response (audio signal down-sampled to 100 Hz) reached 20% of the maximum and remained higher than 20% for at least 50 ms. Noise or slurred words could be incorrectly detected as response time by the algorithm. Therefore, every trial was audio replayed and visually inspected (interactive custom Matlab interface), and manual correction of response time was applied when necessary (using the same 20% of maximum threshold; the procedure was blind to whether the condition was congruent or not, and the position of the stimulus was not known). Such correction allowed us to include slurred responses as long RTs rather than excluding these informative trials as errors (error rate was too low to be analyzed, as indicated in the next paragraph).

#### Statistics

Trials with response times over 2000 ms (0.6%), those for which the subject gave an incorrect response (0.6%) and responses shorter than 250 ms (0.05%, considered as anticipatory responses; only 9 cases between 20 and 52 ms), were excluded from the analyses. The distribution of response times was highly skewed towards long delays. In order to estimate the correct shape of the distribution, we performed 2*2*2 ANOVAs (with factors task, congruency and hemifield) on the transformed data of each subject and inspected the distributions of residuals. Log and Inverse transformations resulted in distributions of residuals reasonably close enough to the Normal distribution, with the best fitting obtained for the Inverse transformation. Furthermore, the inspection of residuals allowed us to verify the absence of outlier values. Notice that not transforming the data while applying classical criteria for the exclusion of outliers (like values above 2 or 3 SD) would have led us removing many lawful data points, meaning measures that actually do belong to the ‘true’ distribution of the data [33]. All analyses were performed on the central tendency of response times measured for each subject and condition (inverse of the mean of inverse response times; results were almost identical if using the median). We computed 95% Confidence Intervals (CI), using Cumming’s ESCI software when necessary [28].

#### Strength of the synesthetic association

**Fig 1:**
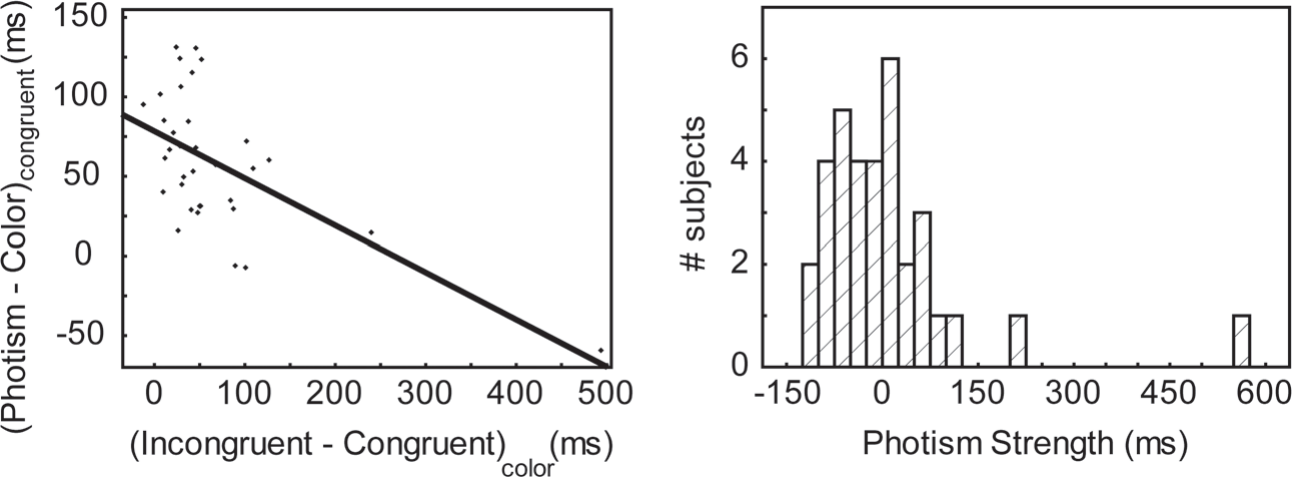
Individual variability for the strength of synesthetic associations. *Left*: Interference for the Color Naming task (x-axis) is negatively correlated with the speed to name synesthetic colors relative to real colors (y-axis). *Right*: The distribution of the index of photism strengh is unimodal, with a few subjects with extreme values. The subject with the largest photism strength could be unambigously characterized as a “projector” based on questionnaires and phenomenological reports, while the subject with the second largest value was characterized as an associator. Several other subjects, with lower values, were tentatively categorized as projectors based on questionnaires. The average effect size of the congruency effect in the color naming task (x-axis of the left panel) is 65 ms (CI = [34 96] ms). If excluding the two large values (above 3 standard deviations), it is 46 ms (CI = [34 59] ms).

Synesthetes form a heterogeneous group: there is much variability in the way they report their synesthetic experiences. It has been proposed that synesthetes should be classified as either “projectors” or “associators” based on their first-person reports, but we and others have found this classification ambiguous and unreliable [16, 34]. Following Flournoy [35], we characterized synesthetes according to the strength of their synesthetic associations. We used a “photism strength” measure similar to that designed in our previous study ([16]; see their Figure 4 for an example). This measure was constructed based on the results of Dixon et al. [25] and Ward et al. [26] (Rothen et al.[36] further showed correlations between the responses to questionnaires and performances to Stroop tasks). These authors had observed that, at the group level, so-called “projectors” and “associators” exhibited a different pattern of results when comparing response times and interference in the photism and color synesthetic tasks. Projectors had stronger interference in the color task than in the photism task (meaning that they had more difficulty ignoring the synesthetic color than the real color), contrary to associators. Moreover, projectors tended to name synesthetic colors faster than real colors. We did not expect individuals to differ for color interference in the photism task (we supposed that color processing was similar), so we did not include in our index the interference in the photism task (mean interference was 103 ms, CI = [86 119] ms). Indeed, interferences in both tasks were not correlated (non-parametric Spearman R = -0.22, N = 34, p = 0.22; Pearson correlation r = -0.26, CI = [-0.55 0.08]; we had previoulsy suggested that this interference term could equalize “for volitional control and speed” [16], which may differ between subjects but be equivalent for different tasks; the absence of correlation shows little support for this assumption). Interference in the color naming task was negatively correlated to the RT to name synesthetic colors, in accord with the results by Dixon et al. [25] and Ward et al. [26] (Fig. 1; Spearman R = -0.49, N = 34, p = 0.003; Pearson correlation r = -0.61, CI = [-0.79–0.34]). Our index of synesthetic strength (‘photism strength’, *ps*) was:

$$ps={\left(Incongruent-Congruent\right)}_{color}-{\left(Photism-Color\right)}_{congruent}$$ (1)

Subtracting the term (Incongruent—Congruent)_photism_ or using effect sizes instead of the central tendency, like we had done in our previous study [16], gave very similar results. A positive *ps* index indicates a strong association between graphemes and synesthetic colors (strong interference by photisms) and an easier task when naming photisms. Since the Color task was always performed before the Photism task the index could be compared between subjects.

## Results

**Fig 2:**
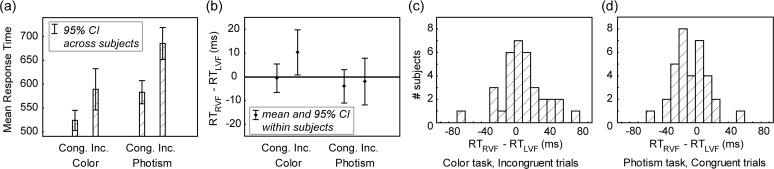
Response times (*RT*) in synesthetic Stroop tasks. Letters and numbers were presented either in the left visual field (*LVF*) or the right visual field (*RVF*). *(a)* Average RT (inverse of the mean inverse RT, mean of LVF and RVF) across 34 synesthetes as a function of Congruency, in the Color and the Photism naming tasks. *Cong*. = congruent colors (graphemes were printed with the color corresponding to the synesthetic color for each synesthete). *Inc*. = incongruent colors. *CI* = confidence interval. *(b)* Within subjects RT differences for stimuli presented in the right and the left hemifields. *(c*,*d)* Hemifield differences across synesthetes for the interference by synesthetic colors (color naming task) *(c)* and naming synesthetic colors *(d)*. All values are within 3 standard deviations of the distributions.

We compared the response times when stimuli were presented within the left or the right visual field, for congruent and incongruent stimuli and in both tasks (Fig. 2 and Table 1).

**Table 1:** Mean response time differences when stimuli were presented in the left or the right hemifield.

stimuli	N	t	p Student	p Wilcoxon	RVF—LVF (mean)	η_p_^2^	95% CI
Color naming task							
congruent	34	−0.21	0.83	0.50	−0.6 ms	0.001	[−7 6] ms
**incongruent**	**34**	**2.2**	**0.03**	**0.02**	**10 ms**	**0.13**	**[0.8 20] ms**
Photism naming task							
**congruent**	**34**	**−1.16**	**0.25**	**0.17**	**−4 ms**	**0.04**	**[−11 3] ms**
incongruent	34	−0.42	0.68	0.74	−2 ms	0.005	[−12 8] ms

The conditions for which we expected hemifield differences are highlighted in bold font. The differences have the expected sign in both cases.

For incongruent stimuli in the color naming task, response times were on average 10 ms longer for stimuli presented in the right than in the left visual field (larger interference by synesthetic colors in the RVF, Fig. 2B and C). We also computed this difference by subtracting the left and right RTs for congruent trials (considered as a baseline: differential effect, corresponding to the interaction effect in the ANOVA). The results were strictly similar (Student t = 2.11, p = 0.04, p(Wilcoxon) = 0.03, η_p_^2^ = 0.12, 95% CI = [0.4 21.4] ms). Synesthetic colors were also 4 ms faster to name for congruent stimuli in the RVF (photism task, Fig. 2, B and D). Both results are compatible with the processing of synesthetic colors preferentially in the left hemisphere.

There was, however, much variability across subjects (Fig. 2, C-D), as reflected by the large confidence intervals for the effect sizes. For the lateralization of the interference by synesthetic colors (color naming task), Cohen’s d was 0.38 (CI = [0.03 0.73]), corresponding to a small effect size (possibly ranging from negligible to medium effect size. Effect sizes are considered as small for 0.2 < d < 0.5 and large for d > 0.8). The difference for photism naming was even weaker.

We tried to explain some of this variability in order to have a better estimate of effect sizes. We first evaluated whether some variability was related to handedness. Two synesthetes were left-handed and one was ambidextrous. Removing them did not improve the estimation: CI = [1.7 22] ms for the interference by synesthetic colors (color naming task), CI = [-11 4] ms for photism naming. Thirty-three synesthetes (including the 3 above) filled out a simplified version of the Edinburgh Handedness Inventory (EHI; subjects had to report whether they prefer to use their right or left hand, or both, for ten usual actions; scores vary between −1 and 1). When restricting the analysis to the 25 strongly right-handed subjects (EHI Score > = 0.8), estimation was less precise for the interference in the color naming task ([-3 21] ms), but improved slightly for photism naming ([−14–0.06] ms, corresponding to Student p = 0.048).

Then, we considered whether lateralization effects were correlated to the strength of synesthetic associations (photism strength index: see Materials and Methods, Equation (1). The construction of the lateralization measure is completely independent of the photism strength measure, even though based on the same data). That was the case neither for the interference in the color naming task (Pearson r = 0.006, CI = [-0.33 0.34], Spearman R = -0.16) nor for photism naming (Pearson r = 0.06, CI = [-0.28 0.39], Spearman R = -0.02). We only observed a weak correlation for the hemifield difference in the color naming task for congruent stimuli (Pearson r = 0.34, CI = [-0.004 0.61], Spearman R = 0.34, p = 0.049), meaning that for synesthetes with strong associations, there could be a conflict between synesthetic and real colors even when colors were similar, when presented on the right side (this is an *ad hoc*, *a posteriori* explanation).

All but one synesthete were presented both letters and digits. While letters and digits are linguistic symbols, digits also convey quantity information. Neuropsychological [37] and neuroimaging studies [38–41] suggest that they might be processed in distinct areas (see Discussion). Lateralization effects of synesthetic colors may occur only for letters, or even in opposite directions for letters and digits. We recomputed the mean response times in each condition independently for letters (34 subjects) and digits (33 subjects). We observed no difference between letters and digits. The lateralization of the interference in the color naming task was on average 8 ms for letters and 10 ms for digits; the lateralization of photism naming was on average −1 ms for letters and −7.5 ms for digits. Confidence intervals were all larger than when estimating response times from both letters and digits.

**Fig 3:**
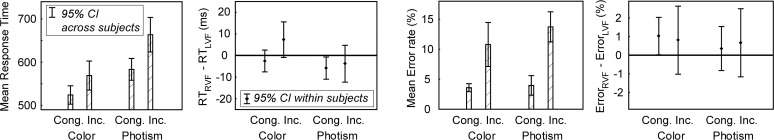
Alternative analysis, considering long RTs as errors. *(a*,*b)* For each subject and condition, the central tendency of the RT distribution was estimated using the mean of the log values, after removing outliers to the distribution (see text). We computed group analyses on the exponential of this mean value. The results are similar to those based on the inverse transformation (no outlier removal), shown in Fig. 2. *(c*,*d)* Outliers were added to the few errors and all were considered as errors for the purpose of the analysis. In contrast with response times, the hemifield differences for error rates were similar in all conditions, with slightly more errors on the right side overall (on average, each subject made one more error on the right side in each experiment; p = 0.03, CI [0.1 1.3] %).

Finally, we wondered whether the measure of the central tendency of the RT distribution best captured the cognitive interference. In Stroop tasks, subjects have to filter out the irrelevant information (real or synesthetic color). We observed that on most trials subjects managed to do it while still responding fast, but on a few trials they seemed to get confused and slowed down or made a mistake. Most subjects did not make many real errors (and not enough to allow their statistical analysis), but on several instances, they started to utter the wrong response before correcting themselves. Because many of these trials were ambiguous, we did not consider them as errors, but we computed the RT as the beginning of the correct response. Most errors were therefore included in our analysis of RT. However, our model assumed the existence of a single distribution. Using the inverse transformation, we did not observe any bimodal or skewed distribution in any subject (we did not have to remove any outlier). But we may apply another strategy, considering that either the filtering mechanism is “on” and the subject responds fast and accurately, or it is “off” and the subject responds slowly or makes a mistake. Therefore, we could consider long RTs as corresponding to failures of the filtering mechanism, analogous to errors. When exploring the distributions of RTs in each subject and experiment, we observed that the Lognormal function produced a good fit for most of the data except for long RTs. As an exploratory analysis, we considered that RTs had a lognormal distribution except when subjects were about to make errors. For each subject and task, we considered as “outliers” values above 1.5 the interquartile distance above the 75% centile of the distribution of the log(RT) measured in the congruent condition (all trials included except 11 trials with anticipatory responses < 250 ms). These outliers were considered as additional errors. The total percentages of errors computed that way were 3.6% and 10.8% in the color naming task (congruent and incongruent trials) and 4.0% and 13.7% in the photism task (Fig. 3). We did not observe any specific hemifield difference (Student t and Wilcoxon tests, both p-values >. 22 and >. 54 for both effects of interest). For the central tendency measures, however, estimated based on the mean of the log(RT) of the remaining trials, the effects were very similar to those observed when using the inverse transformation (Fig. 3; interference in the color naming task, CI = [-0.9 15.6 ms]; photism naming, CI = [-0.7–11 ms]). This analysis confirms that the effects in both conditions are due to a small shift distributed over the whole distribution of response times, an effect compatible with a short additional callosal transfer.

To summarize, we averaged both effect: RT(RVF-LVF)_Incongruent,Color_ (stronger interference, therefore longer RT in the RVF) and—(RT(RVF-LVF)_Congruent,Photism_)(shorter RT in the RVF). These two measures are respectively the second measure of panel 2b (10 ms on average) and the third measure of panel 3b (4 ms on average). Note that both measures were not correlated across subjects (as if sampling independently the same process). Effect size was 7.1 ms, CI [1.4 12.9] ms. If using the log(RT) of trimmed data, it was 6.6 ms, CI [1.6 11.6] ms (Student t and Wilcoxon tests, all p-values between 0.01 and 0.017).

## Discussion

We performed a lateralized version of synesthetic Stroop tasks on 34 subjects. We found some evidence for stronger interference by synesthetic colors and faster naming of synesthetic colors when letters or numbers were presented in the right hemifield. Effect sizes were small (a few milliseconds), as expected if the only difference for stimuli presented on the left side was the additional callosal transfer of the information to the left hemisphere. The 95% confidence interval of the effect was, however, very close to zero, and would therefore need to be replicated in an independent study. If confirmed, this lateralization effect could lead to interesting speculations about the neural bases of synesthesia.

The first question would be whether the lateralization is due to the processing of graphemes or synesthetic colors. Graphemes must be identified and attended to in order for colors to be associated with them [24, 42]. Therefore, the processing of grapheme-color synesthesia could be lateralized because the association of colors to graphemes is lateralized or because grapheme processing is lateralized. While linguistic skills are dominantly processed in the left hemisphere, as reminded in the Introduction, single letters may not be processed the same way as semantic material such as words. James et al. [38] proposed that letters could be processed like any other object of expertise (i.e. bilaterally represented in the ventral pathway) because they do not elicit complex linguistic processing. This view has been formalized under the ‘Linguistic Processing Load (LPL)-based ordering’, which states that only the serial decoding of successive letters requires specific lateralized linguistic processing [43, 44]. The left-lateralized Visual Word Form Area (VWFA, [45]) is indeed specifically activated for letter strings rather than individual letters [46]. In addition, digits might be processed in areas distinct from letter processing [40] and also, possibly, bilaterally [41]. The available neural evidence seems too scarce to conclude yet whether processing of individual letters and numbers is bilateral or lateralized. Behavioral evidence is also scarce and ambiguous. We saw in the Introduction that the left-lateralization of word processing did not necessarily translate to lateralization effects in Stroop tasks [21]. Stroop tasks can, in any case, be performed only with color words, not individual letters or numbers. However, using a simple letter-naming task, Bashore and colleagues [47] measured an LVF rather than an RVF advantage. In summary, letter processing, even if left-lateralized like words, may not lead to hemifield differences in Stroop tasks like those observed here for the synesthetic Stroop. Digits might be processed in areas distinct from letters and possibly bilaterally [41], yet we did not observe any difference between letters and digits. We are currently lacking a precise, validated, model of the different neuronal steps involved in the processing of letters and digits, as well as their association to colors, in order to achieve a proper interpretation of what actually is lateralized in grapheme-color synesthesia. However, until progress is made on that front (that may lead to reconsider our results), we suggest that the present RVF advantage is related more to synesthetic colors than to grapheme identification, and, therefore, that the semantic association of colors to letters and numbers may be coded predominantly in the left hemisphere (especially if graphemes are processed bilaterally).

In order to test this hypothesis, useful control experiments would require testing non-synesthetes on these tasks as well as testing synesthetes on a classic Stroop task. Unfortunately, exact control experiments are not possible. The photism naming task is not possible for non-synesthetes, by definition (a letter naming task is a different task), and there is no interference to measure in non-synesthetes in the color naming task of graphemes. Running the traditional Stroop task on synesthetes is not a good control task because color words also trigger synesthetic colors, leading to a complex pattern of possible interference effects. A potentially interesting experiment, however, could involve training non-synesthetes to associate colors to letters and numbers. Classic Stroop and grapheme naming tasks could be performed before training and serve as baseline. Then, subjects would be trained to associate colors to graphemes until they exhibit Stroop interference effects similar to those observed for synesthetes (e.g. [48]; see [49] for a review) allowing the direct, within subject comparison of semantic lateralization for words and associated colors. Such an experiment could produce two equally interesting outcomes. (1) Trained non-synesthetes could show no lateralization effect in the synesthetic Stroop tasks, unlike synesthetes. This result would strongly link lateralization to the synesthetic experience. Indeed, in most studies, training was successful in inducing automatic letter-color associations but not the synesthetic experience [49]: subjects did not report “seeing colors” on graphemes (synesthetic phenomenology may yet appear sometimes after very extensive training: see [50]). (2) Trained non-synesthetes could show lateralization effects like synesthetes. The control experiments performed before training would help specify whether lateralization is due to grapheme processing or color associations. In the latter case, the result would argue for a strong similarity between trained synesthesia in adults and learned synesthetic associations by children [51, 52], suggesting a continuum between grapheme-color synesthetic associations and normal associations, similarly to the continuum proposed between sequence-space synesthesia and normal visuospatial imagery [53, 54].

## Supporting Information

S1 Dataset Individual data of the 34 subjects. The Excel file contains 5 sheets, corresponding to the different analyses presented in the paper. Each line corresponds to the data of one subject. The order of subjects is the same in all the sheets. For values used in the figures, the name of the figure is indicated on the first line of the corresponding columns.
(XLS)

S2 Dataset Graphemes and colors used for each participant. (The Excel file contains 1 sheet. Each line corresponds to the data of one subject. The order of subjects is the same as in S1 Dataset. Each letter or number is printed in the case used and in red, green, blue or yellow to denote that they were named that way by synesthetes. Actual colors were adjusted for each synesthete on the screen computer used to correspond to their exact synesthetic colors. For other colors, their name is indicated within brackets.
(XLS)
